# Phenotypic Microarrays Suggest *Escherichia coli* ST131 Is Not a Metabolically Distinct Lineage of Extra-Intestinal Pathogenic *E. coli*

**DOI:** 10.1371/journal.pone.0088374

**Published:** 2014-02-07

**Authors:** Abdulaziz Alqasim, Richard Emes, Gemma Clark, Jane Newcombe, Roberto La Ragione, Alan McNally

**Affiliations:** 1 Pathogen Research Group, Nottingham Trent University, Nottingham, United Kingdom; 2 School of Veterinary Medicine and Science, University of Nottingham, Nottingham, United Kingdom; 3 Advanced Data Analysis Centre, University of Nottingham, Nottingham, United Kingdom; 4 Department of Microbial and Cellular Sciences, University of Surrey, Guildford, Surrey, United Kingdom; 5 Department of Bacteriology, Animal Health and Veterinary Laboratories Agency, Weybridge, United Kingdom; 6 School of Veterinary Medicine, University of Surrey, Guildford, Surrey, United Kingdom; University of Hyderabad, India

## Abstract

Extraintestinal pathogenic *E. coli* (ExPEC) are the major aetiological agent of urinary tract infections (UTIs) in humans. The emergence of the CTX-M producing clone *E. coli* ST131 represents a major challenge to public health worldwide. A recent study on the metabolic potential of *E. coli* isolates demonstrated an association between the *E. coli* ST131 clone and enhanced utilisation of a panel of metabolic substrates. The studies presented here investigated the metabolic potential of ST131 and other major ExPEC ST isolates using 120 API test reagents and found that ST131 isolates demonstrated a lower metabolic activity for 5 of 120 biochemical tests in comparison to non-ST131 ExPEC isolates. Furthermore, comparative phenotypic microarray analysis showed a lack of specific metabolic profile for ST131 isolates countering the suggestion that these bacteria are metabolically fitter and therefore more successful human pathogens.

## Introduction

Urinary tract infections (UTIs) are among the most common bacterial infections acquired both in the community and hospital settings [1]. UTIs occur in all age groups and in both genders [2], [3], while their incidence increases with age [4]. Additionally, they are more common in women than men [2], with an estimated 33% of women suffering from a UTI by the age of 24 [5]. UTIs can lead to other severe infections such as bacteraemia, pyelonephritis and sepsis [6]. Worldwide, approximately 150 million people are diagnosed with UTI each year, which has a great impact not only on public health, but also on the global economy [7].

It is well documented that *Escherichia coli* is the main causative agent of UTIs [8], and the extra-intestinal pathogenic *E. coli* [ExPEC] group have the capability of causing community-acquired UTIs, accounting for more than 85% of infections [9]. Over the past decade, ExPEC have shown an increased level of antimicrobial resistance to front-line antibiotics such as trimethoprim and ciprofloxacin, and resistance to these antibiotics has been observed in as many as 20–45% of ExPEC isolates [1], [10]. ExPEC have also been associated with a high level of extended spectrum β-lactamase (ESBL) gene carriage [11], [12]. This is of great concern since it can limit the therapeutic choices used for treating infections, and these organisms may also act as a major reservoir of antimicrobial resistance.

Molecular epidemiological analysis of ESBL-producing ExPEC isolates by multilocus sequence typing (MLST) has recently uncovered the emergence of a prevalent ExPEC clone, namely, *E. coli* sequence type 131 [13]. *E. coli* ST131 is a CTX-M ESBL producing *E. coli* clone [13]. It belongs to the serotype O25:H4, and to the highly virulent phylogroup B2 [14], [15]. *E. coli* ST131 has been implicated as a major cause of dissemination of the CTX-M-15 class of ESBL gene [13]. Although *E. coli* ST131 has been associated with high levels of antimicrobial resistance, it has been suggested to display increased pathogenesis [16], countering the hypothesis that high levels of antimicrobial resistance come at the expense of a fitness advantage, which leads to decreased pathogenesis [1]. Several studies have reported an increased virulence associated gene (VAG) carriage in ST131 isolates [17], and have also found them responsible for causing a range of severity in disease cases [18], [19].

Previous work conducted in our laboratory reported the presence of *E. coli* showing high levels of antimicrobial resistance in a collection of monomicrobial and polymicrobial urine samples [10]. Further characterisation of the isolates found that ST131 was the predominant strain type within the collection, that the ST131 strains were responsible for the high levels of antimicrobial resistance in the collection and that there were variations in VAG profile between strains with no specific VAG profile associated with ST131 [20]. Although the possession of specific virulence factors, such as adhesins and iron acquisition determinants [21], enhances the ability of *E. coli* to cause a UTI, ST131 has not shown a significant difference in its VAG carriage compared to other uropathogenic *E. coli* (UPEC) of different STs such as *E. coli* ST73, ST69 and ST127 [20], [22]. This questions the role of VAG carriage in the current success of ST131 [15], [23], suggesting the presence of other factors that can contribute to ST131 fitness.

Several reports have proposed that bacterial metabolic potential can enhance fitness leading to increased pathogenesis. For example, previous studies have shown that sugar metabolism in enterobacteria [24], and the possession of specific metabolic enzymes [25], may increase bacterial virulence. A recent study comprising 47 biochemical tests using the Vitek2 Advanced Expert System for metabolic profiling on a collection of 300 UPEC isolates concluded that ST131 isolates have higher metabolic potential profiles in comparison to other ST isolates. It also showed that ST131 isolates have a significant association with eight biochemical tests including those for peptidase, decarboxylase, and alkalinisation activity. Moreover, it also found a correlation between metabolic activity and antibiotic susceptibility profiles, with multi-drug resistant isolates showing the highest metabolic potential [26].

In this study, we tested and compared the metabolic activity of a collection of extra-intestinal pathogenic *E. coli* isolates including ST131 and non-ST131 isolates, using all available API metabolic profiling substrates (Biomerieux, UK). We further tested the metabolic activity of ten *E. coli* isolates to a more comprehensive level using Biolog automated phenotypic microarray technology in order to provide a comparison between ST131 and *E. coli* of different STs in terms of their global metabolic potential, and to examine the correlation between the antimicrobial resistance and the metabolic activity of ST131 isolates. Our data agrees with that of Gibreel *et al* (26) in that analysis of a limited number of metabolites can produce differential profiles for different sequence types. However, our Biolog analysis demonstrated no detectable difference in metabolic fitness between ST131 and other ExPEC sequence types, suggesting *E. coli* ST131 does not display elevated metabolic fitness. In addition we reanalysed the genome sequences of a dozen ST131 isolates previously sequenced by our group [27], [28] creating an ST131 core genome which we compared against reference ExPEC genome sequences, showing that there is little difference at the genome level in presence or absence of metabolic operons.

## Materials and Methods

### Bacterial Strains

A collection of fifty *E. coli* isolates, twenty five ST131 and twenty five non-ST131 isolates, were included in the metabolic profiling assay. Thirty six of these isolates were collected between October 2008 and June 2009 from urine samples of elderly patients from Queens Medical Centre, Nottingham, while the other fourteen isolates were collected between March 2011 and June 2011 from urine samples of patients from Queens Medical Centre, Nottingham. The MLST of the isolates and identification of virulence factors and antibiotic susceptibility profiles have been previously described [10], [20], [29]. A subset of ten *E. coli* isolates, five ST131 and five *E. coli* non-ST131, were used to carry out the Biolog phenotypic microarray assay. The five ST131 isolates were previously genome sequenced [27], and varied in CTX-M-15 gene carriage, source of isolation, and invasion levels. The non-ST131 isolates were chosen to represent the major ExPEC STs associated with human disease [20]. Table 1 shows full details of the strains used in the phenotypic microarray assay.

**Table 1:** Details of strains used in the phenotypic microarray assay.

Strain	*E. coli* ST	Strain history	Patient source	CTX-M carriage	Reference
**UTI18**	ST131	UTI	Community	CTX-M-15	[20]
**UTI32**	ST131	UTI	Hospital	CTX-M-15	[20]
**UTI226**	ST131	UTI	Hospital	–	[20]
**UTI570**	ST131	UTI	Community	–	[20]
**UTI587**	ST131	UTI	Community	CTX-M-15	[20]
**UTI396**	ST393	UTI	–	–	[20]
**UTI501**	ST69	UTI	–	–	[20]
**UTI89**	ST95	Uncomplicated cystitis	–	–	[39]
**CFT073**	ST73	Acute pyelonephritis	–	–	[40]
**P5B**	ST10	Bacteraemia	–	–	[41]

### Metabolic Profiling Assay by API Reagents

Four API kits: API 20E, ID32 E, API 50 CH and API ZYM kits (Biomerieux, UK) were used in metabolic profiling. This resulted in a total of 120 biochemical tests and allowed the measurement of carbon source utilisation, carbohydrate fermentation and enzymatic activity of *E. coli* isolates. Preparation of bacterial suspensions and inoculation of test kits were performed according to the manufacturer’s instructions (Biomerieux, UK). The assays were performed in duplicate on two independent occasions giving completely concordant results.

### Biolog Phenotypic Microarray (PM) Assay

The PM assay was performed using Biolog Inc. (Hayward, CA). This assay consisted of two 96 well PM panels (PM1, PM2A), which were used to test the ability of five *E. coli* ST131 and five non-ST131 isolates to utilise 190 carbon sources. *E. coli* isolates were plated out on LB agar (Fisher Scientific, UK) at 37°C prior to starting the assay. The bacterial cell suspension for each isolate was prepared by transferring around 20–25 bacterial colonies into a sterile tube containing 15 ml of sterile dH_2_O. A uniform suspension was made until a turbidity of 42% transmittance (T) ±1% in the Biolog turbidimeter was obtained. Two millilitres of this cell suspension was then added to 10 ml of inoculation fluid-0 (IF-0)+120 µl dye A to yield a final cell density of 85% T. Afterwards, 100 µl of the 85% T cell suspension was added to each well. The plates were then placed in the OmniLog reader (Biolog), and incubated for 48 h. The OmniLog reader analyses the plates every 15 minutes, converting the pixel density in each well to a signal value reflecting cell growth and dye conversion. Phenotypic microarray data analysis was performed using a signal value calculation approach described previously by Homann *et al* [30]. Each substrate was tested in duplicate per strain.

### Statistical Analysis

The production of heat maps for API metabolic profiling results was performed using SPSS PAWS (version 20.0) statistics software. Phenotypic microarray results were analysed using R statistics package. The significance of association between *E. coli* STs and different biochemical tests used for the metabolic profiling assay was determined by performing Fisher’s exact test (FET) in a pairwise fashion, and the threshold for statistical significance was a *P* value of ≤0.05. Testing for correlation between metabolic profile and sequence type was performed by principal component analysis (PCA) in R.

### Comparative Genomics

To compare metabolic potential between strains at a genomic level, an ST131 core genome was created to ensure any differences observed were conserved across all the ST131 strains. This was done as previously described by our group [28] using all available ST131 genomes [27]. The ST131 core genome was then compared against CFT073, UTI89, and P5B (table 1) using ACT [31] in a pairwise fashion to determine metabolic loci uniquely present or uniquely absent to ST131.

## Results

### API Test Results Identify a Small Number of ST131 Discriminatory Tests

**Figure 1:**
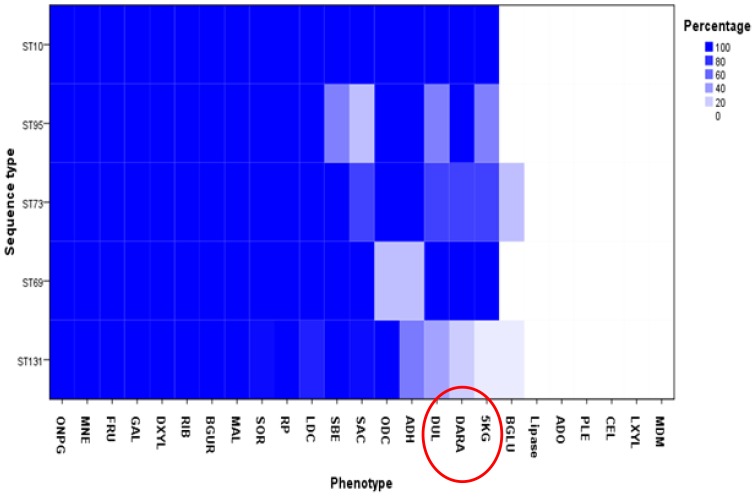
Heat map showing a comparison of results obtained from testing 25 API biochemical substrates on *E. coli* ST131 and four major ExPEC STs representing the whole substrates used in phenotypic profiling assay. Darker shaded areas indicate higher percentage of strains capable of utilizing the substrate. The inability of ST131 to utilise 5KG, Dulcitol (DUL), DARA, cellobiose and esculin is highlighted by red circle.

120 API test reagents were used to perform metabolic profiling on twenty five ST131 and twenty five non-ST131 isolates belonging to the four major ExPEC sequence types: ST69, ST73, ST95 and ST10. Figure 1 shows a comparison of results obtained from selected biochemical tests for all fifty *E. coli* ST131 and non-ST131 isolates. All isolates were positive for utilisation of thirty substrates, and negative for forty three others, with variations between STs in terms of their capability of utilising the remaining forty seven substrates.

ST131 isolates exhibited a strong trend in the inability to utilise five tested substrates: 5-keto-D-gluconate (5KG), D-ARAbinose (DARA), esculin, cellobiose and dulcitol, in comparison to other ST isolates. With regard to 5KG utilisation, only 2 of the 25 ST131 isolates (8%) were able to utilise this substrate, whilst the utilisation percentages for ST69, ST73, S795 and ST10 isolates were 100%, 85%, 66% and 60%, respectively. Additionally, ST131 isolates exhibited poor DARA utilisation compared to other ST isolates. Only 20% of ST131 isolates were able to utilise this substrate compared to 100% for ST69 isolates, 85% for ST73 isolates, 100% for ST95 isolates, and 60% for ST10 isolates. Similarly dulcitol utilisation percentage for ST131 isolates was 36% compared to 100% for ST69 isolates, 85% for ST73 isolates, 83% for ST95 isolates, and 60% for ST10 isolates. The capability of ST131 isolates to utilise esculin was 24% compared to 42% for ST69, 100% for ST73, 66% for ST95 and 100% for ST10 isolates. The cellobiose utilisation percentage for ST131 isolates was 12% compared to 42% for ST69 isolates, 50% for ST73 isolates, 33% for ST95 isolates, and 40% for ST10 isolates.

ST131 isolates did not show higher metabolic activity for any biochemical substrate tested when compared to other ST isolates, which is in contrast to the previous study [26]. The ability of ST131 to utilise substrates for which it was more active in that study, such as ODC, βGUR and SAC, was not higher using API methodology, while some other substrates do not appear in the API substrate panel.

### Biolog Phenotypic Microarray Assay Shows No Significant Difference in Metabolic Capacity between ST131 and Other ST Isolates

**Figure 2:**
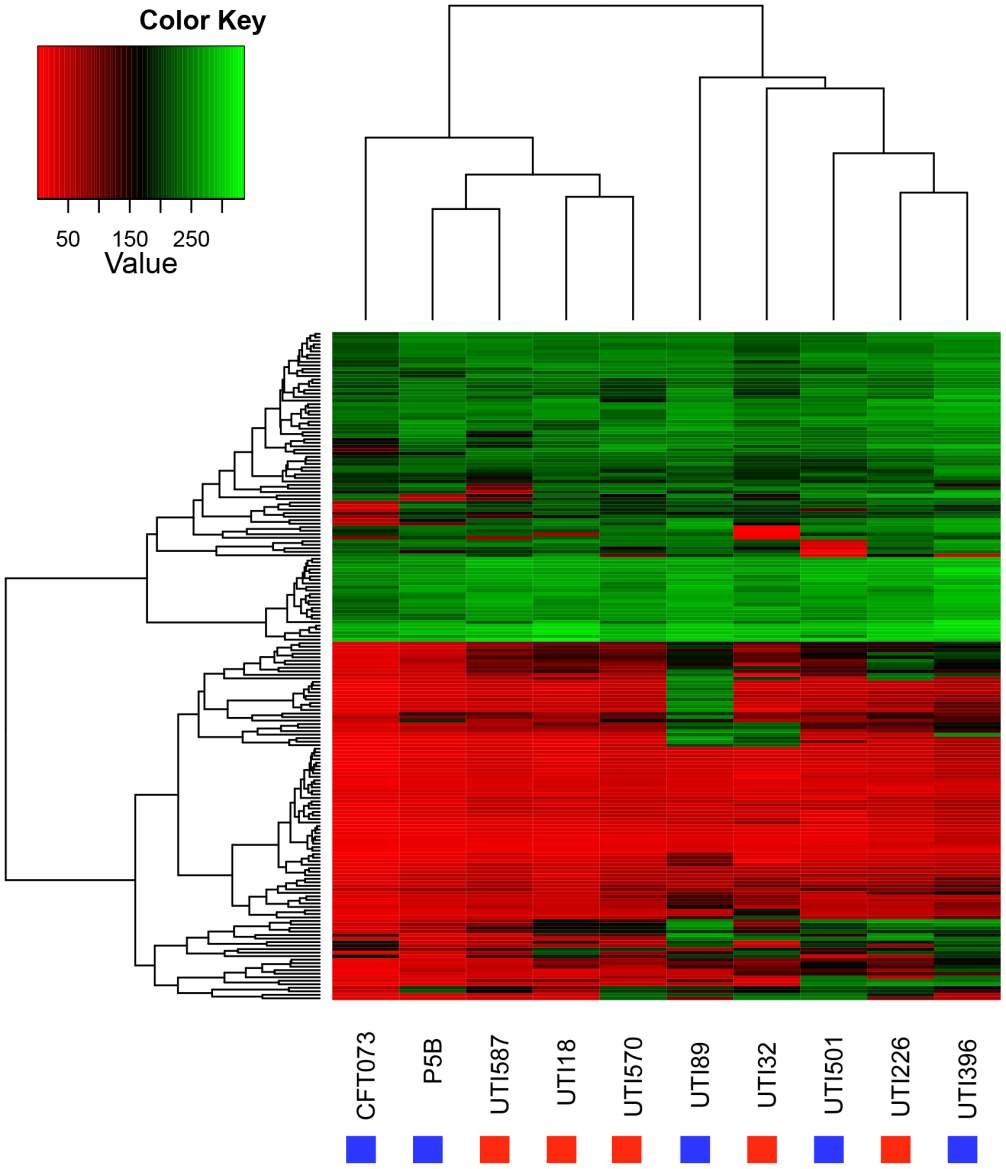
Cluster heat map showing the signal values of *E. coli* ST131 and non-ST131 isolates obtained from 190 biochemical tests using PM assay, with red showing no utilisation through to green showing high levels of utilisation. A UPGMA dendrogram informed by the metabolic profile is presented above the heatmap. ST131 strains are represented by red blocks, and non-ST131 strains by blue blocks.

In light of the contrasting results above with a previous study showing ST131 enhanced metabolic function, we utilised the superior discriminatory power of the phenotypic microarray assay to fully characterise the metabolic capacity of five *E. coli* ST131 and five non-131 isolates. Figure 2 shows a heat map for all metabolites tested, with values corresponding to the intensity of utilisation for the different nutrient sources. There were very few detectable differences in the metabolic capacity between ST131 and other ST isolates for the majority of tested carbon sources. As with the commercial test reagents there were some strain-specific differences in metabolite utilisation but these were not consistent in either ST131 or non-ST131 groups, and ST131 isolates were not associated with a specific metabolic profile.

Considering the metabolic activity of CTX-M-15 producing ST131 isolates, UTI32 showed higher metabolic potential in comparison to other ST131 isolates as its signal values were the highest for a range of substrates such as M-inositol, palatinose, D-tagatose, malonic acid and N-acetyl-L-glutamic acid, L-histidine and L-phenylalanine and putrescine. In contrast it also showed the lowest utilisation levels for other substrates including N-acetyl-D-galactosamine, and signal values for D-raffinose and L-malic acid were lower than for other ST131 and non-ST131 isolates. UTI587 showed a lower potential for utilising the following substrates: dulcitol, glycyl-L-glutamic acid, 3-0-b-d-galacto-pyranosyl and melibionic acid. Additionally, UTI18 had the lowest signal value for α-Keto-butyric acid substrate among ST131 isolates. The ST131 CTX-M-15 negative isolate, UTI226, had the highest metabolic activity among ST131 isolates for pyroglutamic acid, chondroitin sulfate c, β-cyclodextin, amygadin, gentiobiose and D-lyxose carbon sources. In contrast it also had the lowest ability to utilise b-hydroxy-butyric acid compared to other ST131 isolates.

Taken together our data suggests that ESBL gene carriage is not associated with a specific metabolic activity profile within the ST131 isolates, and that variation is on a more generic strain to strain level. With respect to the metabolic activity of non-ST131 isolates, UTI89 exhibited an increased ability to metabolise some carbon sources compared to other isolates, and was the only isolate capable of utilising carbon sources such as tyramine, gelatin, xylitol, G-amino-butyric acid and D,L-octopamine. Additionally, the signal values of UTI89 were higher than that of other isolates for D-arabitol, L-arabitol and 3-methyl glucose.

In short, although there are some differences in the metabolic traits between isolates, these differences are very much strain-specific and are not detected at a sequence type level. The generation of a UPGMA dendrogram based on utilisation of metabolites confirmed this, with the ST131 and non-ST131 strains equally dispersed throughout the dendrogram, suggesting that ST131 are not a metabolically distinct group of ExPEC strains.

### Principal Component Analysis of Phenotypic Microarray Data Set Confirms the Non-existence of an ST131 Metabolic Cluster

**Figure 3:**
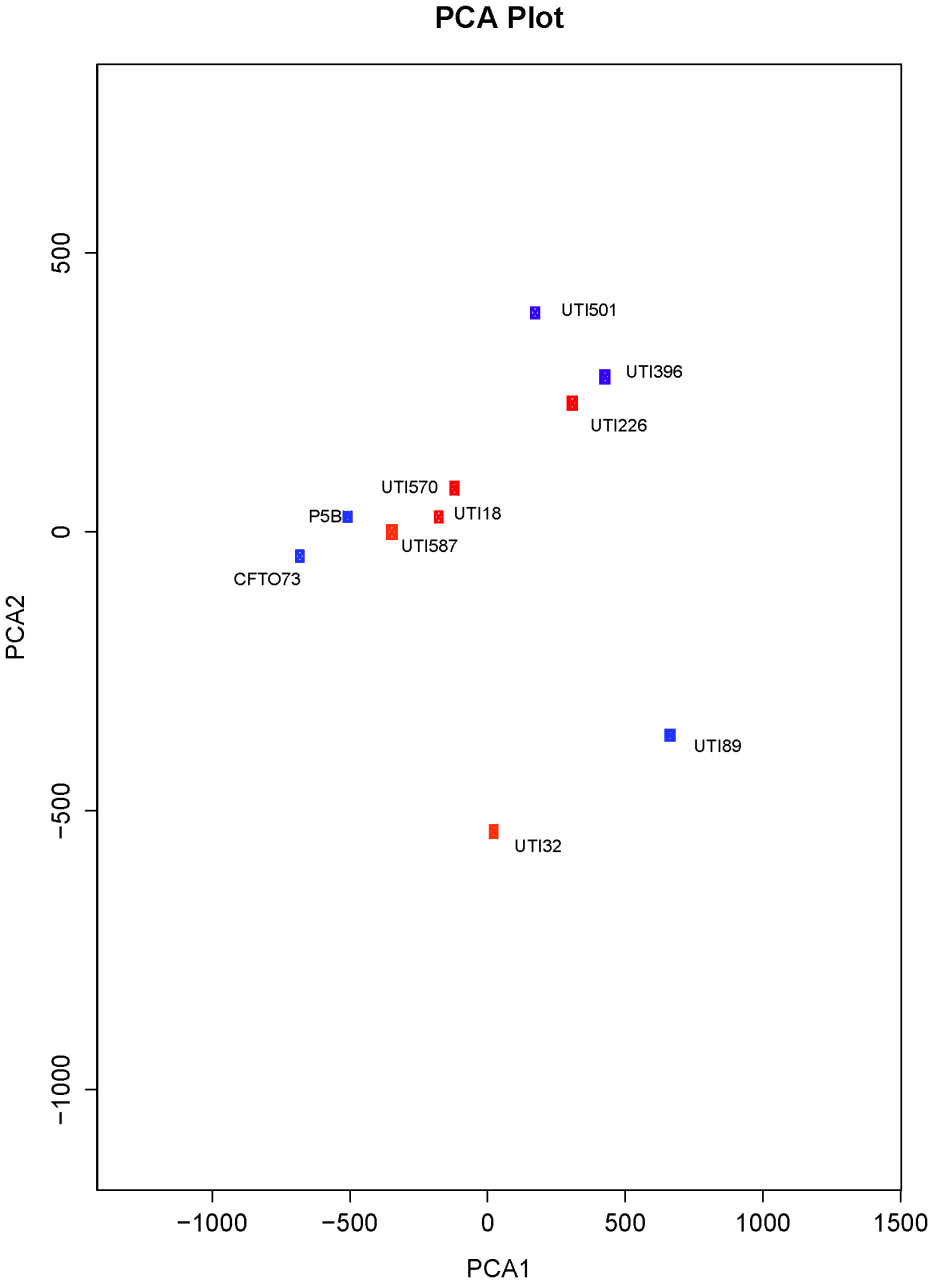
Principal component analysis for metabolic profiles obtained from an analysis of the phenotypic microarray data set. ST131 strains are denoted by red blocks, and non-ST131 strains by blue blocks.

To further confirm that ST131 are not a metabolically distinct group of ExPEC strains, we performed principal component analysis on the phenotypic microarray metabolic profile data set. The PCA1/PCA2 plot (Figure 3) shows clearly that ST131 isolates are not grouped based on metabolic properties, but rather are dispersed throughout the PCA plot amongst the non-ST131 isolates. In conjunction with the heat-map based dendrogram our data suggests that *E. coli* ST131 are not a metabolically distinct clade of ExPEC.

### Comparative Genomic Analysis Confirms the Absence of ST131 Associated Metabolic Loci

Given that the vast majority of the strains analysed by phenotypic microarray in our study have been genome sequenced [27], [28] we sought to further confirm our metabolism observations by correlating them to gene presence/absence data at a whole genome level. We adapted an approach recently used to successfully identify clade specific metabolic functions in *Campylobacter* [32] by constructing an ST131 core genome and then comparing this against the CFT073, UTI89, and P5B genomes (table 1) using ACT [31], concentrating on genes and operons with predicted or confirmed metabolic functions.

**Figure 4:**
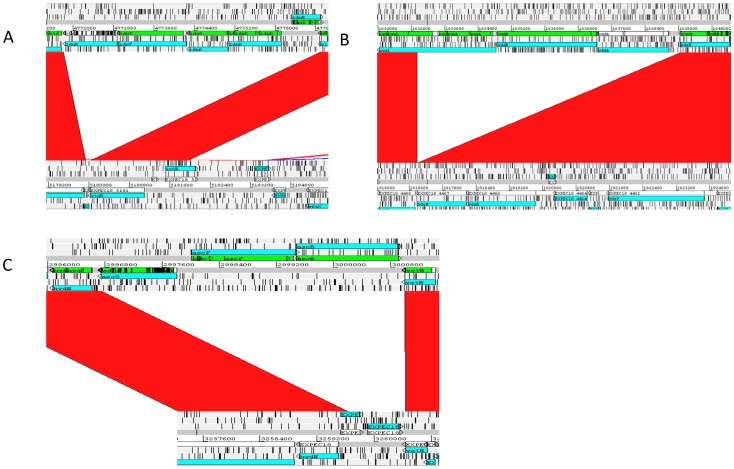
ACT comparisons of the (A) *idn* (B) *ydd* and (C) *asc* loci from CFT073 (upper genome) and the core ST131 genome (lower genome).

Our data showed that the vast majority of core ST131 specific genes, and indeed genes not present in all ST131 were phage, transposon, and IS associated sequences. With respect to metabolism we found only 3 clear differences in the *idn, ydd,* and *asc* operons, which we have previously reported [27]. These three operons are involved in utilisation of two of the five metabolites (5KG and cellobiose) identified as being under-utilised in ST131 strains by API. The *idn* operon is a subsidiary pathway for the gntII gluconate metabolism system, and the *ydd* A and B genes are transporters for the gntI gluconate system, whilst the *asc* operon encodes a combined arbutin/salicin/cellobiose uptake and metabolism pathway. Figure 4 shows ACT comparisons of the three loci from CFT073 and the core ST131 genome. Gene content differences relating to the other three differential metabolites could not be found. Therefore our data suggests that presence or absence of loci does not correlate well with metabolic profile. This indicates that the subtle differences that have been observed in metabolism of substrates in ST131 compared to other ExPEC may not be clearly distinguishable at a genome level and may be down to discreet mutations in other upstream or downstream metabolic pathways which then impinge on metabolism of the identified differential substrate.

## Discussion

Metabolism is an important factor in bacterial colonisation of any given environment, and in particular of animal and human hosts. It is suggested that bacteria may need an extensive metabolic repertoire to accommodate changes in pH and nutrient availability that result from their transition from the environment to a within-host niche [33]. Several studies have demonstrated the role of metabolism in enhancing the colonisation and virulence of *E. coli*. For example, the increased capability of the *E. coli* CFT073 strain to catabolise the amino acid D-serine during UTI can lead to increased levels of colonisation and virulence gene expression [34]. Similarly a recent study has shown that enterohaemorrhagic *E. coli* (EHEC) uses fucose to modulate its virulence and metabolic genes [35].

A recent study focussed on the metabolic potential of *E. coli* ST131, the most commonly isolated cause of extra-intestinal *E. coli* infections world-wide and a recently emerged clone of ExPEC associated with multi-drug resistance [26]. The study examined the ability of ST131 drug resistant and sensitive isolates to utilise 47 biochemical substrates in comparison to non-ST131 ExPEC strains, using the Vitek2 Advanced Expert System for metabolic profiling. In total 300 UPEC isolates were compared, with the study concluding that ST131 isolates have higher metabolic potential in comparison to other ST isolates on the basis of their ability to utilise more of the substrates than non-ST131 isolates. The study showed a significant association between ST131 isolates and utilisation of eight biochemical tests including those for peptidase, decarboxylase, and alkalinisation activity. Additionally the study also described a correlation between metabolic activity and antibiotic susceptibility profiles, with ESBL carrying, multi-drug resistant isolates showing the highest metabolic potential [26].

In the study we report here, we tested and compared the metabolic activity of a collection of extra-intestinal pathogenic *E. coli* isolates including ST131 and non-ST131 isolates, employing another commonly used commercial metabolic profiling system (Biomerieux, UK). Our data agrees with that of Gibreel *et al* in that analysis of a limited number of metabolites can produce differential profiles for different sequence types. Our study showed reduced metabolic capacity of ST69 to utilise ODC, and of ST95 to utilise SAC. With respect to ST131 our data showed lower metabolic activity for five substrates, namely 5-keto-D-gluconate (5KG), D-ARAbinose (DARA), esculin, cellobiose and dulcitol in comparison to other major ExPEC STs. Some of these findings are in direct agreement with the Gibreel study which also demonstrated a significant negative association between ST69 and ODC, between ST95 and SAC, and between ST131 and 5KG [26]. However the major discussion point of the Gibreel study, that ST131 are more metabolically flexible and “fit” is in disagreement with the data produced using API biochemical tests. Of the Vitek positive association tests which also feature in the API test panel (ODC, βGUR and SAC) none showed higher levels of metabolism in ST131 isolates compared to non-ST131 ExPEC. The most likely explanation for this is due to differences in the way substrate utilisation is measured between the fully automated Vitek system and the API test strip method. The validity of this as a potential explanation for the discrepancy is highlighted by comparison of our results for dulcitol utilisation in ST131 when measured by API and by phenotypic microarray. Our phenotypic microarray data showed very little difference in dulcitol utilisation between ST131 and other ST isolates, in complete contrast to the API data. This is almost certainly due to the fact that the PM plates are used to measure carbon source oxidation and not sugar fermentation as in the API test [36]. Therefore we suggest that different utilisation capabilities between strains could be observed depending on the principle of assay used to determine the metabolic activity.

Using a total of forty seven biochemical tests on three hundred isolates the Gibreel study concluded ST131 strains are metabolically more flexible. However our data of one hundred twenty biochemical tests on fifty isolates is suggestive of ST131 strains having slightly reduced metabolic potential. In an attempt to further determine the metabolic profile of ST131 *E. coli* in comparison to other ExPEC, we compared ten isolates using Biolog phenotypic microarray technology, five ST131 and five non-ST131 ExPEC. Phenotypic microarrays have been utilised to study the metabolic flexibility of various bacterial species [37] and provides the highest level of resolution currently available for the metabolic capacity of cells. Heat map visualisation of our data indicates there is no distinct metabolic signature for *E. coli* ST131 and that any differences in metabolic repertoire are at an individual strain level rather than by sequence type grouping. This is further supported by a principal component analysis of the microarray data, showing there is no clustering of ST131 based on metabolic repertoire. An obvious caveat to our findings is the limited number of strains examined. However our comparison is of five ST131 strains, all of which have previously been shown to be genetically homogeneous and phylogenetically clustered with the EC985 and NA114 genome sequenced strains [27], [28]. Therefore if ST131 were a metabolically distinct clade of ExPEC with enhanced metabolic potential we would expect to see some form of clustering of our 5 isolates by principal component analysis of the phenotypic microarray data.

Given that the genome sequences of almost all of the strains subjected to phenotypic microarray are available we sought to contextualise our metabolism findings at a whole genome level. We created an ST131 core genome as previously described by our group [28] the rationale being that any ST131-discriminating metabolic repertoire would have an accompanying genetic signature associated with the ST131 clade. This was then compared to the genomes of CFT073, UTI89, and P5B. Our data failed to uncover any substantial differences in the metabolic gene repertoire of ST131 compared to the three other ExPEC genomes. However, when we focussed on operons responsible for the 5 discriminatory tests identified by API (5-keto-D-gluconate (5KG), D-ARAbinose (DARA), esculin, cellobiose and dulcitol) we could find genetic deletions that would account for only two of those phenotypes, in the *idn* and *ydd* operons involved in 5KG metabolism and the *asc* operon involved in cellobiose uptake. Our data appears to suggest that differences in metabolic repertoire between bacteria cannot simply be mapped to genome data and gene presence or absence. It is likely that small mutations in pathways complexly linked to the metabolic function observed could result in knock on effects which would be extremely difficult to pinpoint and associate with the phenotype under investigation.

In conclusion, it is our opinion that the previous study of *E. coli* ST131 metabolism performed by Gibreel et al [26] excellently identifies metabolic markers which could have enormous importance in rapid identification or selection of ST131 isolates in human samples. In our study, we concurred that the use of a limited number of biochemical tests can produce differential profiles for different sequence types. Our phenotypic profiling data supports this as we found clear differences in the metabolic activity between ST131 and non-ST131 strains for 5 of 120 biochemical substrates. However, when we comprehensively tested the global metabolic repertoire of a limited number of *E. coli* strains using the phenotypic microarray system, we found no difference in the overall metabolic fitness between ST131 and non-ST131 isolates. In addition, comparison of genomic data also suggested very little difference in the repertoire of metabolic gene loci between ExPEC sequence types.

The current ST131 literature seems highly confused with respect to the reasons behind the success of the *E. coli* ST131 clone. Early PCR based studies attributed this to the combination of phylogenetic group B2 and the presence of specific virulence factors such as pathogenicity island marker (*malX*), outer membrane protein (*ompT*) and uro-pathogenic specific protein (*usp*) being more common among ST131 than in other CTX-M producing *E. coli* strains, and suggested that these factors might play a major role in the worldwide dissemination of ST131 [13]. Another study suggested that the rapid global spread of the CTX-M-15 producing *E. coli* might be due to the acquisition of the IncFII plasmids which are associated with harbouring many antimicrobial resistance genes [38]. Johnson and colleagues have demonstrated that undefined phylogenetic group B2-associated factors may provide a fitness advantage to ST131 [19], whilst the recent paper on which our work was based suggested ST131 strains were more metabolically active [26].

Our previous work describing the genetic homogeneity of ST131 [27] combined with the data presented here provide yet another confounding hypothesis that *E. coli* ST131 do not display altered metabolic fitness to other closely related ExPEC from a global metabolic and genomic perspective. An obvious caveat to both our findings and those of Gibreel *et al* is that different strain sets have been compared using different methodologies, and indeed this may be applicable to many of the dichotomous results in the literature regarding ST131. We propose that a co-ordinated international effort to fully understand the evolutionary mechanisms behind the emergence of *E. coli* ST131 is now imperative in order to combat this most serious of bacterial pandemics, and of future episodes of novel *E. coli* lineage emergence.
